# Evaluation of molecular typing for national surveillance of invasive clinical *Haemophilus influenzae* isolates from Denmark

**DOI:** 10.3389/fmicb.2022.1030242

**Published:** 2022-11-17

**Authors:** Hans-Christian Slotved, Thor Bech Johannesen, Marc Stegger, Kurt Fuursted

**Affiliations:** Department of Bacteria, Parasites and Fungi, Statens Serum Institut, Copenhagen, Denmark

**Keywords:** Denmark, capsular genes, genotyping, serotyping, *Haemophilus influenzae*

## Abstract

Ha*emophilus influenzae* is a gram-negative coccobacillus known to cause respiratory and invasive infections. It can possess a polysaccharide capsule that can be categorized into six different serotypes (i.e., Hia, Hib, Hic, Hid, Hie, and Hif) and non-encapsulated strains that are defined as non-typeable. Furthermore, *H. influenzae* can be characterized into eight biotypes (I–VIII). Traditionally, isolates have been serotyped and biotyped using phenotypic methods; however, these methods are not always reliable. In this study, we evaluate the use of whole-genome sequencing (WGS) for national surveillance and characterization of clinical Danish *H. influenzae* isolates. In Denmark, all clinical invasive isolates between 2014 and 2021 have been serotyped using a traditional phenotypic latex agglutination test as well as *in silico* serotyped using the *in silico* programs “hinfluenzae_capsule_characterization” and “hicap” to compare the subsequent serotypes. Moreover, isolates were also biotyped using a phenotypic enzyme test and the genomic data for the detection of the genes encoding ornithine, tryptophan, and urease. The results showed a 99–100% concordance between the two genotypic approaches and the phenotypic serotyping, respectively. The biotyping showed a 95% concordance between genotyping and phenotyping. In conclusion, our results show that in a clinical surveillance setting, *in silico* serotyping and WGS-based biotyping are a robust and reliable approach for typing clinical *H. influenzae* isolates.

## Introduction

*Haemophilus influenzae* is a gram-negative coccobacillus that can cause respiratory infections as well as invasive infections such as septicemia and meningitis (Nørskov-Lauritsen et al., 2021). It is categorized into six serotypes (i.e., Hia, Hib, Hic, Hid, Hie, and Hif) according to the polysaccharide capsule, as well as non-encapsulated strains that are referred to as non-typeable *H. influenzae* (non-cap) (Potts et al., 2019). In the pre-vaccine era, *H. influenzae* serotype b (Hib) was a frequent cause of bacterial meningitis among young children (Bijlmer, 1991; Nørskov-Lauritsen, 2014; Jalalvand and Riesbeck, 2018), but after the introduction of Hib vaccines in the 1980s, the numbers diminished dramatically worldwide (Morris et al., 2008; Nørskov-Lauritsen, 2014). The Hib vaccine was introduced into the Danish immunization program in 1993 as a part of the diphtheria-tetanus-pertussis-polio combination vaccine recommended at 3, 5, and 12 months of age, with high vaccination coverage of 96% (12-month vaccination; ^1^ accessed 17 December 2021), and it has been estimated to have an effectiveness of more than 97% for the prevention of Hib-related meningitis (^2^ accessed 29 November 2021).

*Haemophilus influenzae* has traditionally been serotyped by phenotypic methods and PCR (Potts et al., 2019; Watts and Holt, 2019). Recently, two *in silico* approaches using whole-genome sequencing (WGS), “hinfluenzae_capsule_characterization” (Potts et al., 2019) and “hicap” (Watts and Holt, 2019), have been described that allow easier serotyping.

*Haemophilus influenzae* can furthermore be characterized based on eight biotypes defined by the presence and absence of the three enzymes, namely, tryptophanase (indole production), urease, and ornithine decarboxylase (Nørskov-Lauritsen, 2014).

In this study, we evaluated the use of two different *in silico* serotyping programs based on WGS for national surveillance of invasive clinical *H. influenzae* isolates from Denmark received between 2014 and 2021. Additionally, we investigated the clonal relatedness to serotype and biotype.

## Materials and methods

### Clinical isolates

Since October 2007, surveillance of invasive Hib infections in Denmark has included mandatory submission of isolates to the Neisseria and Streptococcus Reference Laboratory (NSR) at Statens Serum Institut (SSI) following an executive order (BEK nr 1102 of 20/09/2007), and although the surveillance specifies only Hib, the reference laboratory receives the majority of invasive *H. influenzae* isolates from all Danish regional laboratories of clinical microbiology.

All isolates were from invasive cases from mainland Denmark. An invasive case was defined as the presence of invasive *H. influenzae* in a patient who had a positive culture result for *H. influenzae* from cerebrospinal fluid (CSF), blood, or other normally sterile sites.

**FIGURE 1:**
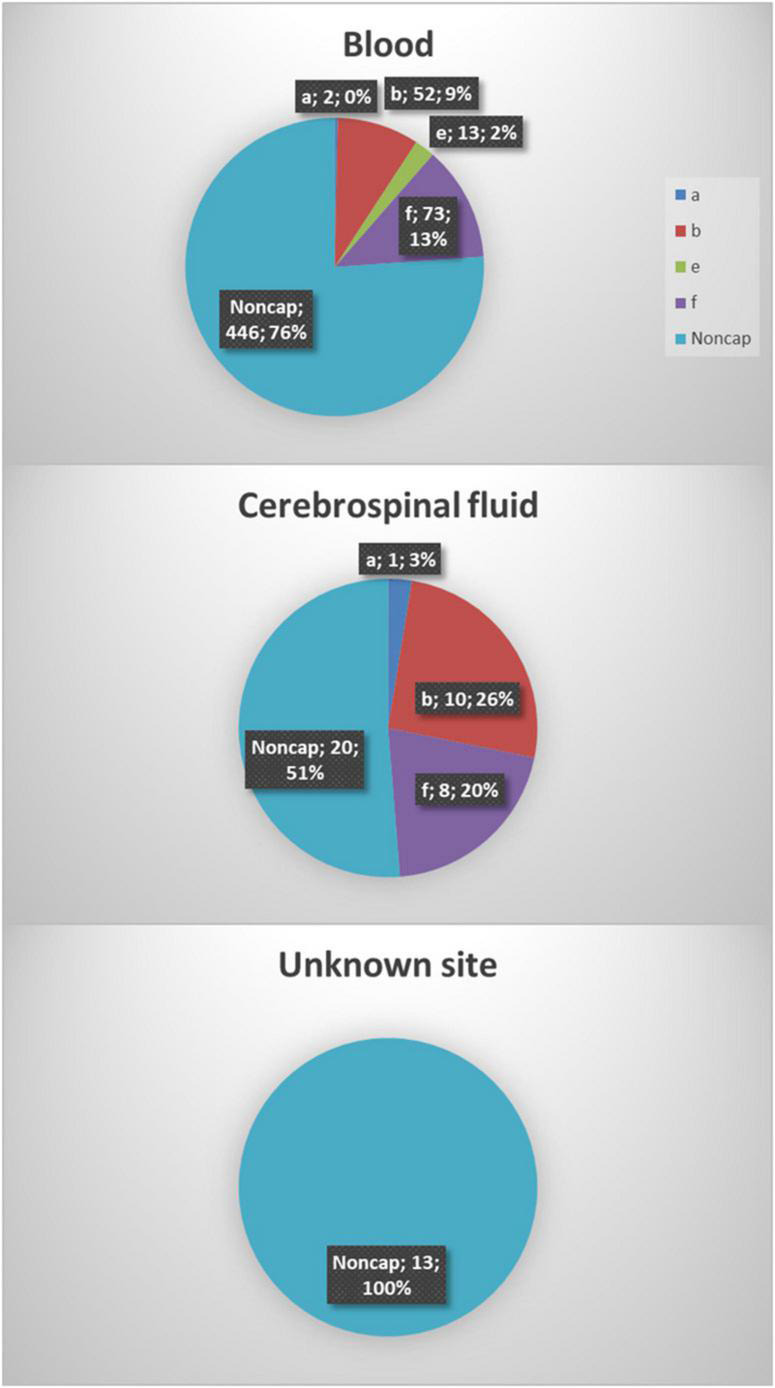
A total of six hundred thirty-eight *Haemophilus influenzae* isolates were included in the study. The majority of the isolates were from blood samples (91.8%, 586), 6.1% (39) were from cerebrospinal fluid, and information on the origin of 2% (13) of isolates was not available.

Data on serotypes and sample sites on all clinical *H. influenzae* isolates between 2014 and 2021 were retrieved from the Danish laboratory surveillance system at the Neisseria and Streptococcus Reference Laboratory (Figure 1). The WGS and phenotyping were performed on isolates consecutively when received during the study period. All isolates were grown on chocolate plates for both DNA preparation and phenotyping.

### Species identification of *Haemophilus influenzae* isolates

The identification of *H. influenzae* was performed as previously described (Kilian, 1976; Fuursted et al., 2016). Briefly, species identification was performed on strains transferred directly from bacterial colonies using matrix-assisted laser desorption/ionization time-of-flight mass spectrometry (MALDI-TOF MS) (Bruker Daltonics; Compass 1.4, Version 3.4, Build 3.4.76.0). Species identification was based on the standard MALDI-TOF score value [Biotyper database version MBT 6903 MSP Library (#1829023)] and confirmed with WGS data using KmerFinder.^3^

### Phenotypic biotyping

From 2014 to 2019, the *H. influenzae* isolates were typed phenotypically into eight biotypes based on their variable production of tryptophanase (indole production), urease, and ornithine decarboxylase (ODC), as described in other studies (Kilian et al., 1979; Nørskov-Lauritsen, 2014). Phenotypic biotyping was not performed on the 2020–2021 isolates.

### Serotyping

The isolates were serotyped using latex agglutination test (SSI Diagnostica, Denmark) specific for *H. influenzae* covering all six known serotypes (i.e., Hia, Hib, Hic, Hid, Hie, and Hif).

### Whole-genome sequencing and assembly

Whole-genome sequencing was performed as previously described (Fuursted et al., 2016; Slotved et al., 2021). Briefly, genomic DNA was extracted using a DNeasy Blood & Tissue Kit (QIAGEN, Hilden, Germany), and fragment libraries were constructed using a Nextera XT Kit (Illumina, San Diego, CA, USA) followed by either 150 or 250 bp paired-end sequencing on either the MiSeq or NextSeq 550 platform (Illumina, San Diego, CA, USA), respectively, according to the manufacturer’s instructions. The paired-end data were *de novo* assembled using the SKESA assembler (SKESA version 2.2) (Souvorov et al., 2018).

The genomic sequence data for the 638 clinical isolates have been deposited at the European Nucleotide Archive (ENA) under project no. PRJEB56415.

### Genotyping

Genotyping was performed on all isolates using two *in silico* serotyping programs (Potts et al., 2019; Watts and Holt, 2019) with default parameters. The version used for the *in silico* program “hinfluenzae_capsule_characterization” was from the GitHub site: https://github.com/Vikash84/hinfluenzae_capsule_characterization, accessed 07-10-2022. The version used for the *in silico* program “hicap” was the version hicap 1.0.3: https://anaconda.org/bioconda/hicap, accessed 07-10-2022.

For isolates with deviations in phenotype/genotype, the genotype was additionally verified using BLASTN (BLAST 2.9.0 + version) using the sequences as described by Maaroufi et al. (2007) against the assembled genomes.

For the molecular detection of the biotype genes, partial sequences were used from the *H. influenzae* reference genome Rd KW20 (GenBank accession ID L42023); gene HI_0590 (1308 bp), gene HI_1389.1 (1434 bp), and gene HI_0535 (786 bp).

### Multilocus sequence typing and phylogenetic analysis

Multilocus sequence typing was performed by uploading the assembled genomes to the PubMLST database (see text footnote 3, accessed 08-02-2022) to identify sequence types (ST) and corresponding clonal complexes (CC) for all isolates. STs sharing at least six of seven allelic variants were grouped into CCs (Shabayek and Spellerberg, 2018).

A phylogenetic tree was created based on single-nucleotide polymorphisms (SNPs) detected in the core genome of the isolate collection. SNPs were identified using NASP (Sahl et al., 2016) with BWAmem for mapping against the chromosome of *H. influenzae* isolate Rd KW20 (GenBank accession ID L42023), and GATK was set to remove positions with less than 10-fold depth and 90% unambiguous variant calls after removal of duplicated regions in the reference using NUCmer. The resulting SNP matrix was purged for recombination using Gubbins (Croucher et al., 2015). The phylogenetic tree was generated using a maximum-likelihood approach with IQ-TREE (Nguyen et al., 2015)^4^ and ModelFinder as implemented with 100 bootstraps before visualization using iTOL version 6 (Letunic and Bork, 2021).

### Ethical considerations

The data and samples from patients were collected routinely for national surveillance purposes; therefore, no ethical approval or informed consent from patients or guardians was required. The study was approved by the Danish Data Protection Agency (record number 2007-41-0229). For further details on SSI’s permission to present and publish epidemiological data, see^5^ accessed 30-06-2022,^6^ accessed 30–06–2022). All presented data were anonymized.

## Results

### Characterization of the clinical isolates

Between 2014 and 2021, eight hundred seventy-five invasive *H. influenzae* isolates were received, and 638 (72.9%) of those isolates had both phenotypic and genotypic available data and were included in the study. All serotypes were represented except for serotypes c and d. One reference strain for Hic and two for Hid were included in the testing of capsular genes but not included in the clinical data (Table 1). The majority of the isolates were from blood cultures (91.8%), whereas 39 (6.1%) were from cerebrospinal fluid samples. No clinical information was available for the remaining 13 (2.0%) isolates (Figure 1).

**TABLE 1:** List of isolates with divergent phenotype/genotype for serotype or biotype and missing values.

Isolate number	Phenotype	The *in silico* program “hicap” (Watts and Holt, 2019)	The *in silico* program “hinfluenzae _capsule _characterization” (Potts et al., 2019)	Biotype	Biotype (Genotype)	Biotype (Genotype retest)^c^	MLST
HINF-2014-1112	f	f*	Non-cap [f backbone: fcs2 fragmented (82.66% cov)]	I	I	ND	124
HINF-2015-1428	a	a*	Found genes for serotypes a,b, possible contamination^b^	II	II	ND	2053
HINF-2016-1709	Non-cap	Non-cap	Non-cap	III	II	II	46
HINF-2016-1715	f	f	f	II	I	I	124
HINF-2017-1785	e	e	E	I	V	I	386
HINF-2017-1788	Non-cap	Non-cap	Non-cap	III	VII	II	134
HINF-2017-1789	Non-cap	Non-cap	Non-cap	II	VII	II	57
HINF-2017-1793	Non-cap	Non-cap	Non-cap	II	VIII	II	199
HINF-2017-1802	Non-cap	Non-cap	Non-cap	III	II	II	142
HINF-2017-1808	Non-cap	Non-cap	Non-cap	III	VII	II	145
HINF-2017-1815	Non-cap	Non-cap	Non-cap	III	VIII	III	146
HINF-2017-1828	a	a*	Found genes for serotypes a,b, possible contamination^b^	II	VII	II	56
HINF-2017-1838	Non-cap	Non-cap	Non-cap	III	II	II	422
HINF-2017-1840	f	f	f	I	IV	I	124
HINF-2017-1851	Non-cap	Non-cap	Non-cap	II	VIII	II	136
HINF-2017-1917	e	e	e	III	IV	IV	18
HINF-2018-1932	Non-cap	Non-cap	Non-cap	IV	III	III	165
HINF-2018-1948	b	b	b	I	II	II	709
HINF-2018-1979	Non-cap	Non-cap	Non-cap	III	II	II	183
HINF-2018-1986	Non-cap	Non-cap	Non-cap	II	VIII	II	389
HINF-2018-1987	Non-cap	Non-cap	Non-cap	III	II	II	411
HINF-2018-2029	a	a*	Found genes for serotypes a,b, possible contamination^b^	III	II	II	2057
HINF-2018-2048	b	b*	Non-cap [b backbone: bexB fragmented (89.85% cov)]	I	I	I	Novel
HINF-2019-2063	f	f	f	II	I	I	124
HINF-2019-2079	Non-cap	Non-cap	Non-cap	IV	III	III	165
HINF-2019-2094	Non-cap	Non-cap	Non-cap	I	II	II	103
HINF-2019-2107	Non-cap	Non-cap	Non-cap	VII	V	V	1238
HINF-2019-2113	Non-cap	Non-cap	Non-cap	IV	I	I	Novel
HINF-2019-2195	Non-cap	Non-cap	Non-cap	I	II	II	3
HINF-2019-2210	Non-cap	Non-cap	Non-cap	II	III	III	107
HINF-2021-0022	Non-cap	Non-cap	Non-cap	ND	V	V	210
HINF-2021-0033	f	f*	Non-cap [f backbone: fcs1 fragmented (94.34% cov)]	ND	I	I	124
HINF-2021-0050	e	e*	e	ND	IV	IV	18
HINF-2021-0067	e	e*	e	ND	I	I	386
HINF-NML-07-01	d	d*	Found genes for serotypes d,e, possible contamination^b^	ND	IV	I	47
HINF-NML-21-007	c	c*	Found genes for serotypes f,c, possible contamination^b^	ND	II	II	9
HINF-REF4914	d	d*	Found genes for serotypes d,e, possible contamination^b^	ND	IV	I	47

ND, not done. *Confirmed using the PCR sequences from Maaroufi et al. (2007). ^b^Contamination as defined by *in silico* 2 (Potts et al., 2019). ^c^Using a coverage of 85% and an identity of 85%.

The overall material included 159 (24.9%) typeable and 479 (75.1%) non-typeable isolates (refer to Tables 2, 3).

**TABLE 2:** A total of eight hundred seventy-five invasive cases of *Haemophilus influenzae* were reported in Denmark from 2014 to 2021.

2014–2021	Phenotype	Genotype
**Serotype**		
A	3	3
b	62	62
c	0	0
d	0	0
e	13	13
f	81	81
Non-cap	479	479
Total	638 (72.9%)	638 (72.9%)

Of these, 638 isolates were tested both with phenotypic and molecular serotyping methods according to the *in silico* program “hicap” (Watts and Holt, 2019).

**TABLE 3:** A total of seven hundred and thirteen invasive cases of *Haemophilus influenzae* were reported in Denmark from 2014 to 2019.

2014–2019	Phenotype	Genotype
**Biotypes**		
I	165	162
II	209	218
III	117	111
IV	13	13
V	21	23
VI	4	5
VII	2	1
VIII	0	0
Total	533 (74.8%)	533 (74.8%)

Of these, 533 isolates were tested both with phenotypic and molecular methods.

### Serotyping of the isolates

Across the collection, 638 (100%) and 632 (99.1%) isolates showed concordance between the phenotype and the *in silico* approaches developed by Watts and Holt (2019) and Potts et al., 2019; Tables 1, 2. One serotype b and two serotype f isolates were typed as non-cap isolates, while three isolates were labeled as contaminated due to matches against capsular genes for several *H. influenzae* serotypes (refer to Table 1). The Hic and Hid reference isolates were correctly identified with the Watts and Holt *in silico* method, while the Potts et al. *in silico* method described them as contaminated although detecting possible capsular genes of serotype c and serotype d, respectively (Table 1).

In our reference laboratory, the Watts and Holt *in silico* method was chosen as the reference method. Therefore, we did not perform a second WGS test of all the isolates (six isolates in total) showing contaminating results with the Potts et al. *in silico* method (Potts et al., 2019) (Table 1). This might have improved the typing results of the six isolates when using the Potts et al. (2019) *in silico* method.

### Biotyping

The most common biotypes were biotype II (39%), I (31%), and III (22%), but all biotypes except for biotype VIII were observed. Of the 533 isolates (2014–2019) tested for both phenotype and genotype, 506 (94.9%) isolates showed concordance, while 27 (5.1%) isolates showed divergent biotypes (Tables 1, 3).

**FIGURE 2:**
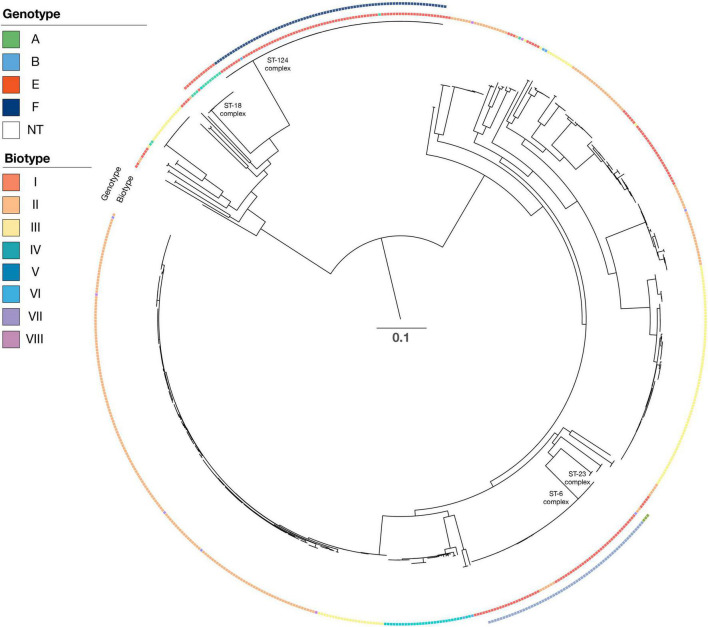
Phylogenetic tree of all isolates. For each isolate, data are presented for the serotype (genotype with color), biotype (biotype with color), and clonal complex. Isolate Hinf Rd KW20 (GenBank nb. L42023) was used as a reference strain in the SNP alignment. Scale bar indicate substitutions per side.

By lowering the coverage and identity to 85 and 85% when blasting for biotype sequences, it was possible to obtain complete concordance for both phenotype and genotype for eight of the 17 isolates with divergent biotypes (Table 1). Unfortunately, we were not able to retest the phenotype of the remaining 19 isolates, and we could therefore not rule out the possibility of diverting the biotype due to laboratory mistakes. The biotypes were not linked to a specific serotype, and each biotype was found to cover both capsular and non-capsular isolates (Figure 2).

### Multilocus sequence typing types and phylogenetic tree

The capsular *H. influenzae* (a–f) were all distributed among six different clonal complexes (Table 4) as follows: ST23 complex (ST56, ST2053, and ST2057), ST6 complex (ST6, ST95, ST190, ST206, ST709, and ST1448), ST18 complex (ST18, ST122, and ST386), and ST124 complex (ST124, ST598, and ST1739).

**TABLE 4:** Sequence type and clonal complex found among the *Haemophilus influenzae* isolates with a capsule.

ST	Clonal complex	A	B	C	D	E	F	Non-cap
56	ST-23 complex	1						0
2053	ST-23 complex	1						0
2057	ST-23 complex	1						0
6	ST-6 complex		21					0
95	ST-6 complex		1					0
190	ST-6 complex		28					0
206	ST-6 complex		1					0
709	ST-6 complex		6					0
1448	ST-6 complex		1					0
Novel	ST-6 complex		4					0
9	ST-7 complex			1				0
47	ST-10 complex				2			0
18	ST-18 complex					8		0
122	ST-18 complex					1		0
386	ST-18 complex					2		0
Novel	ST-18 complex					2		0
124	ST-124 complex						76	0
598	ST-124 complex						1	0
1739	ST-124 complex						4	0

The dominant ST types were ST6 (21 isolates), ST190 (28 isolates), and ST709 (six isolates), all of serotype b. ST18 consisted of 13 serotype e isolates, whereas ST124 consisted of 76 serotype f isolates. Finally, the four ST1739 were all serotype f. The reference strain serotype c belonged to the ST7 complex (ST9), and the two serotype d isolates belonged to the ST10 complex (ST47).

The *H. influenzae* non-cap isolates had 131 different STs, including 40 novel STs. The dominant STs for non-cap were ST103 (n = 29), ST12 (n = 15), and ST159 (n = 13), whereas novel STs were observed for 40 non-cap isolates (Table 5). None of the non-cap isolates were found to be clonal nor related phylogenetically to any of the capsular *H. influenzae* isolates.

**TABLE 5:** Sequence types for *Haemophilus influenzae* non-cap isolates.

ST	Non-cap	ST	Non-cap	ST	Non-cap	ST	Non-cap	ST	Non-cap	ST	Non-cap
2	2	146	2	266	5	567	3	1041	1	1850	1
3	10	147	8	276	4	582	2	1054	1	1904	2
11	4	155	7	311	1	589	1	1069	1	2031	2
12	15	156	1	334	2	597	5	1076	1	2156	1
13	1	159	13	348	2	608	1	1144	1	2332	2
14	5	160	3	349	2	634	3	1170	1	2333	2
34	5	161	1	351	2	652	6	1198	1	2519	1
41	6	165	12	367	10	653	1	1202	1	Novel	40
43	3	176	1	368	3	690	1	1215	1		
46	1	180	2	388	5	697	4	1218	4		
57	10	183	5	389	3	804	1	1220	1		
84	7	187	1	393	5	835	5	1238	1		
98	1	196	1	408	4	836	4	1379	1		
103	29	199	6	409	2	838	1	1382	1		
105	7	200	2	411	3	841	1	1401	2		
107	12	201	2	422	2	914	1	1426	1		
113	2	203	3	425	12	925	1	1497	2		
134	11	208	1	427	2	932	1	1521	1		
136	2	210	4	436	4	943	1	1524	2		
139	7	241	1	472	6	946	3	1591	2		
142	4	245	4	474	2	949	1	1714	1		
143	7	249	1	485	1	958	1	1727	1		
145	11	253	1	524	1	990	1	1773	1		
		262	1	531	2	995	2	1780	2		
		264	1	556	2	1034	11	1834	1		

Phylogenetic analysis of all isolates (Figure 1) shows that all clinical isolates with a capsule clustered both according to serotype and clonal complex. The biotype did not show any type-specific clustering.

## Discussion

The characterization of *H. influenzae* isolates has traditionally been based on phenotypic methods (Kilian, 1976; Kilian et al., 1979; Nørskov-Lauritsen, 2014). With the introduction of the Hib vaccine four decades ago, the importance of *H. influenzae* capsular serotype distribution has increased due to continued monitoring of vaccine efficacy (Satola et al., 2007).

Serotyping of *H. influenzae* isolates has for many years mainly been based on slide agglutination serotyping that detects the expressed capsule (Satola et al., 2007; Potts et al., 2019); however, the slide agglutination serotyping is not always considered reliable, and false-positive Hib designations have been reported (Maaroufi et al., 2007; Satola et al., 2007; Nørskov-Lauritsen, 2014; Potts et al., 2019). With the description of the capsular gene sequences and the development of molecular typing methods, new PCR and WGS-based procedures for *H. influenzae* serotyping have been presented (Maaroufi et al., 2007; Satola et al., 2007; Nørskov-Lauritsen, 2014; Potts et al., 2019; Watts and Holt, 2019). In the study by Potts et al. (2019), 675 invasive isolates were collected from surveillance programs from 27 states in the United States, and 13 isolates of other origins were compared between the *in silico* program “hinfluenzae_capsule_characterization” described serotypes and the serotype results obtained by both PCR and slide agglutination tests. They found that the WGS serotyping method was 99.9% concordant with the slide agglutination tests and completely concordant with RT-PCR. Watts and Holt (2019) tested the *in silico* program “hicap” using 41 publicly available isolate WGS sequences. In 40 of the 41 isolates (98%), they obtained the correct serotype. The difference between the two *in silico* approaches is that the *in silico* program “hicap” uses Prodigal (Hyatt et al., 2010) to identify open reading frames (ORFs) in genomic assemblies and BLAST (Camacho et al., 2009) to identify capsule genes based on a custom reference database. The serotype is then predicted based on the detection of capsule genes. The approach by Potts et al. (2019) performs BLAST directly on the genome assemblies and then parses the identified cap genes for truncations and internal stop codons before predicting a serotype, rather than using external software for gene prediction.

This study, representing 73% of all clinical isolates from 2014 to 2021 from Denmark, found 100% concordance between the capsular genotype and the phenotype (slide agglutination test). This is comparable to the observations by Potts et al. (2019) and Watts and Holt (2019). This is the first study to show that *in silico* serotyping programs are feasible in a national *H. influenzae* surveillance program with the notion that the Hib vaccine efficacy depends on the phenotypic expression of the Hib polysaccharide and not the presence of the capsular sequences.

As a complementary approach to capsular serotyping, a biotyping setup has been described by Kilian in 1976 (Kilian, 1976; Kilian et al., 1979; Nørskov-Lauritsen, 2014). The system is based on eight biotypes defined by variations in the production of tryptophanase (indole production), urease, and ornithine decarboxylase (ODC) (Nørskov-Lauritsen, 2014). However, because the biotype is not related to the capsular serotype and therefore not considered an important part of the efficacy monitoring of the Hib vaccine in Denmark, phenotypic biotyping has not been performed since 2019. However, comparing the phenotypic biotype with the molecular biotype obtained from isolates sampled from 2014 to 2019 did show a high concordance between phenotypic and molecular biotyping. In total, 27 isolates (5.1%) showed diverging biotypes. Adjusting the percentage of coverage and identity could improve the concordance between phenotypic and molecular biotyping (Table 1). However, there was a mixture of capsular *H. influenzae* isolates and non-cap *H. influenzae* isolates among each of the specifically detected biotypes (Figure 2).

While the isolates expressing a capsule could be linked to a specific CC, this was not the case for the non-cap *H. influenzae* isolates, which exhibited a broad diversity of ST types (Tables 4, 5, and Figure 2). Although the ST types in this study could be used to differentiate the capsular *H. influenzae* isolates from the non-cap isolates, it has previously been shown that MLST is not optimal for the identification of capsular *H. influenzae* isolates (Potts et al., 2019). However, we found that the MLST type can indicate the correct identification of the genotype because the capsular isolates appear to be part of only six clonal complexes (Table 4). Therefore, this can, in a national routine *H. influenzae* surveillance program for monitoring the effect of the Hib vaccine, be used as an additional confirmation of correct capsule identification, in that non-cap ST types belonging within these six CC or capsular isolates not belonging to these six CC, will need further evaluation before final capsule reporting.

Based on the results of this study, we recommend a workflow using genotyping either based on PCR (Maaroufi et al., 2007; Satola et al., 2007) or WGS (Potts et al., 2019; Watts and Holt, 2019). Because of the use of the Hib vaccine, we additionally suggest confirming the expression of the Hib capsule using a latex agglutination test for vaccine surveillance. As an additional verification of the serotype, we propose MLST typing as clinical capsular isolates appear to cluster in specific clonal complexes (Tables 4, 5), although the MLST itself cannot be used for serotype identification (Potts et al., 2019).

The weakness of this study is that by only looking at clinical isolates, the tested isolates representing all six serotypes are not equally distributed but dominated by serotypes f, b, and e (Table 2), while only a limited number of isolates with serotype a were detected, and none with serotype c and serotype d. The strength of the study is that the evaluation is based on clinical isolates received from Danish regional laboratories of clinical microbiology, and all the tested isolates showed a strong correlation between the results obtained with genotyping vs. phenotyping, in line with observations in other studies (Potts et al., 2019; Watts and Holt, 2019).

In conclusion, our study showed that there is a complete concordance between phenotypic serotyping and molecular-based methods for Danish clinical *H. influenzae* isolates. The concordance is 100% for both the described *in silico* serotyping methods, replacing laborious phenotypic methods with WGS-based *in silico* approaches in a clinical setting. In addition, biotyping shows a high concordance between molecular and phenotypic methods. Furthermore, in our Danish routine laboratory where epidemiological surveillance of *H. influenzae* for monitoring the effect of the Hib vaccine is an important routine task, the MLST type can be used as an indication of whether an isolated expresses a capsule or not. In that, all capsular-defined isolates in this study belonged to one of six clonal complexes depending on their capsule.

## Data availability statement

The datasets presented in this study can be found in the online repository at the European Nucleotide Archive (ENA) under project no. PRJEB56415.

## Author contributions

H-CS and KF designed the study, analyzed the data, and drafted the manuscript. All authors performed the genomic analyses and reviewed the data, contributed to the manuscript, and critically revised the manuscript, and have approved the final manuscript.

## Footnotes

1
https://statistik.ssi.dk//sygdomsdata#!/?vaccination=3&sex=3&landsdel=100&xaxis=Cohort&show=Graph&datatype=Vaccination

2
https://www.ssi.dk/vaccinationer/boernevaccination/sygdomsforekomst-foer-og-efter-vaccination

3
https://cge.food.dtu.dk/services/KmerFinder/

4
http://www.iqtree.org/

5
https://en.ssi.dk/research

6
https://en.ssi.dk/about-us
